# Fabrication and characterization of scaffolds containing different amounts of allantoin for skin tissue engineering

**DOI:** 10.1038/s41598-021-95763-4

**Published:** 2021-08-09

**Authors:** Yeganeh Dorri Nokoorani, Amir Shamloo, Maedeh Bahadoran, Hamideh Moravvej

**Affiliations:** 1 Mechanical Engineering Department, Sharif University of Technology, Tehran, Iran; 2 Skin Research Center, Shahid Beheshti University of Medical Sciences, Tehran, Iran

**Keywords:** Biomedical engineering, Biological techniques, Biotechnology

## Abstract

Using the skin tissue engineering approach is a way to help the body to recover its lost skin in cases that the spontaneous healing process is either impossible or inadequate, such as severe wounds or burns. In the present study, chitosan/gelatin-based scaffolds containing 0.25, 0.5, 0.75, and 1% allantoin were created to improve the wounds’ healing process. EDC and NHS were used to cross-link the samples, which were further freeze-dried. Different in-vitro methods were utilized to characterize the specimens, including SEM imaging, PBS absorption and degradation tests, mechanical experiments, allantoin release profile assessment, antibacterial assay, and cell viability and adhesion tests. The results indicated that the scaffolds’ average pore sizes were approximately in the range of 390–440 µm, and their PBS uptake amounts were about 1000% to 1250% after being soaked in PBS for 24 h. Around 70% of the specimens were degraded in 6 days, but they were not fully degraded after 21 days. Besides, the samples showed antibacterial activity against *S. aureus* and *E. coli* bacteria. In general, the MTT cell viability test indicated that the cells’ density increased slightly or remained the same during the experiment. SEM images of cells seeded on the scaffolds indicated appropriate properties of the scaffolds for cell adhesion.

## Introduction

Skin, the human beings’ largest organ covering almost all of their body, protects the internal tissues and organs against external damage^1,2^. Therefore, it is often exposed to thermal, physical, microbial, or chemical potential harms^3^. If any of these potentially harmful factors disrupt the skin’s normal structure and function, a wound occurs^4,5^. Based on the skin layers involved (epidermis, dermis, and hypodermis are three main layers of the skin^6^), the wounds can be classified into three categories: (1) superficial wounds, in which only the first layer of the skin, the epidermis, is damaged, (2) partial-thickness wounds, in which both the epidermis and the dermis are impaired, and (3) full-thickness wounds, in which the hypodermis is also damaged in addition to the epidermis and the dermis^4^.

Wound healing is a complex biological process including five stages: (1) hemostasis, (2) inflammation, (3) migration, (4) proliferation, and (5) maturation or remodeling^7^. The natural wound healing process of the body is able to recover minor wounds; however, in case of partial- or full-thickness wounds, this healing process is limited and may not be successful in regenerating a functional skin tissue^8^. The gold standard to treat such severe cases is split-skin grafting (SSG), which involves debriding the patient’s wound and autologous skin grafting^9,10^. However, due to this method’s limitations, such as graft rejection, patient’s donor site pain and morbidity, and infection, other methods have been introduced, among which regenerative medicine has attracted a lot of attention^11^.

Scaffolds play a vital role in many regenerative medicine and tissue engineering approaches and are able to restore and improve the functions of tissue by providing a suitable substrate for cells to grow, proliferate and differentiate^12^. Scaffolds can be created in several types, such as fibrous scaffolds, hydrogel scaffolds, acellular scaffolds, composite scaffolds, and porous scaffolds^12^. Among these different scaffold types, porous scaffolds are widely used for skin tissue engineering because of their unique characteristics, such as good mechanical properties and 3D porous structure encouraging cell penetration^13^. A skin scaffold should be biocompatible and allow the dermal cells to attach, proliferate, and differentiate to restore the skin’s structure and functions^14^. It should also provide a barrier against infection and external damages and possess appropriate mechanical properties and degradation rate^11,14^.

Among various biomaterials, natural polymers have been vastly used to construct skin scaffolds due to their outstanding properties, including but not limited to biodegradability, high biocompatibility, and ability to mimic the extracellular matrix to guide the migration and organization of the cells during the wound healing process^4,11,15^. Chitosan (Ch) and Gelatin (G) are two of the most popular natural biopolymers for fabricating skin scaffolds used in the present study.

Chitosan, a deacetylated derivative of chitin, increases granulation during the proliferation stage of wound healing^16,17^. It is highly biocompatible, has controllable biodegradability based on its degree of deacetylation (DD), and its structure resembles glycosaminoglycans^18,19^. Besides, chitosan is an antibacterial agent against several Gram-negative and Gram-positive bacteria, and its antimicrobial mode of action has been studied^20^. Chitosan also promotes platelet adhesion, which makes this biomaterial a hemostatic agent^21^. Gelatin, obtained by collagen’s denaturation, has several properties making it suitable for skin tissue engineering applications^22^. It is biocompatible, biodegradable, and cost-effective^23,24^. Gelatin is also a hemostatic agent and is suitable for cell adhesion due to Arg-Gly-Asp (RGD) sequences in its structure^12,25^.

In many studies in the field of skin tissue engineering, the scaffold is used for drug delivery to the wound area. In the present study, allantoin is used to improve the wound healing process. Allantoin (All) was first reported to be beneficial for the wound healing process in Macalister’s article in 1912, as mentioned in Ref.^26^. Robinson et al. studied the effect of 0.5% w/v allantoin to treat chronic non-healing wounds in 1935 and concluded that allantoin stimulates wound healing by improving local granulation^26^. Araújo et al. studied the wound healing process affected by allantoin and observed that allantoin enhanced the healing by modulating the inflammatory response and stimulating the proliferation of fibroblasts and ECM synthesis, leading to a more organized skin tissue^27^. Yaşayan et al. investigated the properties of chitosan/collagen wound dressings containing allantoin and lidocaine hydrochloride and concluded that these dressings were biocompatible, improved wound healing process, and may affect patients’ compliance positively^17^. Madrazo-Jiménez et al. observed that their topical chitosan gel containing allantoin and chlorhexidine improved the wound healing process^28^.

Sakthiguru et al. investigated chitosan/gelatin/allantoin films’ characteristics as wound dressings and concluded that allantoin improved the dressings’ antibacterial properties^29^. In this paper, the authors fabricated chitosan/gelatin films with different ratios of gelatin to chitosan and added a specific amount of allantoin to their biocomposite films. To the authors’ knowledge, although allantoin has been used in treating wounds since over a century ago, utilizing it in a porous 3D skin scaffold has not been studied yet. In the present study, a chitosan/gelatin porous 3D scaffold has been fabricated using the freeze-drying method, and EDC/NHS has been utilized to cross-link the samples. Four different amounts of allantoin have been added to the scaffolds, and their in-vitro properties have been investigated. Studying the effect of various amounts of allantoin on chitosan/gelatin scaffolds is another novel item investigated in the present study.

## Materials and methods

### Materials

Chitosan (medium molecular weight, 75–85% degree of deacetylation), gelatin (from pork skin), NHS, Lysozyme enzyme, and Tetrazolium dye for MTT test were purchased from Sigma Aldrich Company. Regarding the product information of lysozyme from chicken egg white for Molecular Biology (Catalog Number L7651, Sigma-Aldrich, USA), Lysozyme activity was ≤ 40,000 units/mg protein. EDC was purchased from BioBasic Inc. (Canada). Allantoin was dedicated by Pars Hayan Co.

### Scaffolds preparation

To prepare the scaffolds, polymer solutions of Chitosan 4%w/v (dissolved in double distilled water, containing 2%v/v Acetic acid) and Gelatin 10%w/v (dissolved in double distilled water) were obtained. Afterward, equal amounts of the two polymer solutions were mixed to obtain the solution containing 2%w/v Chitosan and 5% w/v Gelatin. Four different allantoin concentrations were added to the Ch/G solutions to fabricate the final samples, including 0.25, 0.5, 0.75, and 1%w/v, which are safe for skin protection according to the USA Food and Drug Administration (FDA)^30^. The abbreviated names for these samples are 0.25%All, 0.5%All, 0.75%All, and 1%All, respectively, which will be used in the rest of the paper.

After mixing the final solutions, EDC and NHS (0.3% w/v) were added to cross-link the gels. The cross-linked gels were then kept at − 20 °C overnight, followed by soaking in NaOH 1%w/v solution for 1 h to neutralize them. After washing the samples with double distilled water for another 1 h, the specimens were kept in − 20 °C overnight, followed by freeze-drying for 24 h.

### Scaffolds characterization

#### Morphology and porosity assessment

Scanning Electron Microscopy (SEM, Seron AIS2300C, Korea) was utilized to investigate the scaffolds’ morphology. Coating the freeze-dried specimens with gold was performed prior to observing their surface through SEM imaging.

After preparing the SEM images, the average diameter of the pores and the porosity of the scaffolds were evaluated by Image-J software. The results are presented as average $\pm$ standard error.

Since measuring porosity using SEM images is not an accurate method for analyzing scaffolds’ porosity, the ethanol displacement technique was also employed. To this end, samples were first prepared in a cylindrical shape. Their dry weights (${W}_{0}$) were measured immediately after freeze-drying, and their volumes were also calculated (${V}_{1}$) by measuring their heights and diameters. The hydrogels were then immersed in ethanol 96% v/v at room temperature until being saturated (about 1 h). Subsequently, their weights (${W}_{1}$) were measured following the removal of excess ethanol from the sample surfaces using filter papers. The porosity was then calculated according to Eq. (1). Note that ρ is a constant representing the density of ethanol. The ethanol displacement test was performed three times per sample, and its results are presented as average $\pm$ standard error.

1 $$Porosity \left(\%\right)= \frac{{W}_{1}-{W}_{0}}{\rho {V}_{1}}\times 100.$$

#### PBS absorption behavior

The swelling ratio, PBS uptake, and PBS content of the scaffolds were measured to investigate their fluid absorption behavior. To determine the scaffolds’ swelling ability, the samples were soaked in PBS and weighed in determined intervals to calculate their swelling ratio, according to Eq. (2).

2 $$SR \left(\%\right)= \frac{{W}_{x}-{W}_{0}}{{W}_{0}}\times 100.$$

In this equation, SR, ${W}_{x}$, and ${W}_{0}$ represent swelling ration, the sample’s wet weight in the determined time, and the sample’s dry weight, respectively.

To investigate the ultimate capacity of the scaffolds for PBS absorption, the PBS uptake test was done. To this end, the scaffolds were soaked in PBS, and their wet weights were measured after 24 h. The PBS uptake was calculated based on Eq. (3), in which ${W}_{1}$, and ${W}_{0}$ represent the sample’s wet weight after soaking in PBS for 24 h and the scaffold’s initial dry weight, respectively.

3 $$PBS \,uptake \left(\%\right)= \frac{{W}_{1}-{W}_{0}}{{W}_{0}}\times 100.$$

Furthermore, after being soaked in PBS, the scaffolds’ PBS content was calculated according to Eq. (4), using the measured features of the PBS uptake experiment.

4 $$PBS \,content= \frac{{W}_{1}-{W}_{0}}{{W}_{1}}\times 100.$$

The mentioned experiments were done three times for each specimen, and the results are presented as average $\pm$ standard error.

#### Gel fraction

After fabricating the scaffolds, their gel fraction quantities were measured using the following experiment. First, the dry weights of the scaffolds were measured (${W}_{0}$) and the specimens were soaked in double-distilled water for 24 h. Then, the samples were put in − 20 $^\circ{\rm C}$ following by freeze-drying for 24 h. Finally, the dry weights of the twice freeze-dried samples were measured (${W}_{2}$). The gel fraction of the specimens was calculated using Eq. (5). This test was performed three times per sample, and the results are reported as average $\pm$ standard error.

5 $$Gel\, fraction \left(\%\right)= \frac{{W}_{2}}{{W}_{0}}\times 100.$$

#### Apparent density

The apparent density of a scaffold means calculating its density considering its porosity, which can be obtained by dividing the sample’s dry weight ($W$) by its volume. In this study, the cylindrical samples’ volume is calculated by measuring their heights ($H$) and diameters ($D$) carefully. The apparent density of the scaffolds ($\rho$) is obtained using Eq. (6). The apparent density experiment was carried out three times for each specimen, and its results are presented as average $\pm$ standard error.

6 $$\rho \left(\mathrm{g}/{\mathrm{cm}}^{3}\right)= \frac{W}{\pi \times {\left(\frac{D}{2}\right)}^{2}\times H}\times 100.$$

#### Degradation rate

The scaffolds’ degradation rate is measured in both phosphate buffer saline (PBS) and PBS containing lysozyme enzyme in the present study. First, PBS is prepared by dissolving 8 g NaCl, 0.2 g KCl, 1.44 g Na_2_HPO_4_, and 0.24 gr KH_2_PO_4_ in 800 ml double-distilled water, then adjusting the solution’s volume to 1L. Second, to measure the degradation rate of the samples (initial dry weight: ${W}_{0}$), they were put in PBS at an incubator with a temperature equal to 37 $^\circ{\rm C}$. Then at intended time intervals, the scaffolds were taken out of PBS and put in an oven (55 $^\circ{\rm C}$) to be completely dried. By measuring their weight afterward ($W$), the degradation rate of the specimens could be calculated using Eq. (7).

7 $$Degradation\, rate \left(\%\right)= \frac{{W}_{0}-W}{{W}_{0}}\times 100.$$

Furthermore, the experiment was repeated using PBS containing 0.2 mg/ml Lysozyme to investigate this enzyme’s effect on the samples’ degradation rate. The degradation tests were performed three times per sample, whose results are reported as average $\pm$ standard error.

#### Uniaxial tensile test

The tensile mechanical characteristics of the scaffolds were tested using an H10KS Hounsfield Universal Mechanical Testing Machine. To do this experiment, the specimens were prepared in rectangular shapes (4 $\times 1\times T$ ${\mathrm{cm}}^{3}$; T is the thickness of the samples measured by vernier caliper). The specimens were soaked in PBS for 30 min before the test, followed by removing the excess water from their surface using filter papers. The uniaxial tension experiment was done with a 1 mm/min deformation rate until the samples rupture. The engineering stress ($\sigma$) and strain ($\varepsilon$) (Eqs. (8), (9), respectively) were calculated using the measured force and deformation by the machine.

8 $$\sigma \left({\text{MPa}}\right)= \frac{F}{W\times h},$$

9 $$\varepsilon \left(\%\right)= \frac{\Delta l}{{l}_{0}}\times 100.$$

In the above equations, $F$, $W$, $h$, $\Delta l$, and ${l}_{0}$ represent the force (N), width (mm), initial thickness (mm), the amount of length deformation, and initial length, respectively. This test was done three times for each sample, and its results are presented as average $\pm$ standard error.

#### Rheological behavior

To investigate the scaffolds’ viscoelastic properties, the rheological test was done using an Anton Paar oscillatory rheometer (Physica MCR301). The specimens were soaked in PBS for 30 min prior to the test. Before the main experiments, the scaffolds’ linear viscoelastic (LVE) region was obtained by strain sweep test. Then, the frequency sweep test was carried out to measure the storage modulus (G′) and the loss modulus (G′′) of the scaffolds.

#### Allantoin release profile

The release kinetics of the allantoin from the hydrogel substrates was studied using a spectrophotometer (Hach, DR 5000). For this purpose, the hydrogels were immersed in PBS solution (pH 7.4) and then incubated at 37 °C.

As the first step, the absorption spectrum of a particular concentration of allantoin in PBS solution (pH 7.4) was obtained by adjusting the spectrophotometer at the survey scan mode, which showed a specific absorption peak at 210 nm for allantoin. Then the standard curve of allantoin at 210 nm was obtained.

To study the allantoin release profile from the scaffolds, the absorbance of allantoin released from the samples in PBS solution was measured at 210 nm via spectrophotometer set at ATC mode at determined time intervals, and the relevant concentration of the released drug was calculated based on the standard curve. The buffer solution was replaced at each time point after measurement.

To investigate the allantoin release mechanism from the specimens, the Korsmeyer-Peppas equation was used, as indicated in Eq. (10)^31^. In this equation, ${M}_{t}$, ${M}_{\infty }$, $K$, and $n$ are the amount of allantoin released from the scaffold, the total amount of allantoin present, the kinetic constant, and the diffusion exponent, respectively.

10 $$\frac{{M}_{t}}{{M}_{\infty }} = K{t}^{n}.$$

The allantoin release test was performed three times for each sample, and the results are shown as average $\pm$ standard error.

#### Antibacterial assessment

The samples’ antibacterial activity was evaluated by measuring the inhibition zone around the scaffolds after putting them in contact with two different bacteria. *Staphylococcus aureus* (*S. aureus*), a gram-positive bacterium, and *Escherichia coli* (*E. coli*), a gram-negative bacterium, were chosen for this experiment.

For this test, the bacteria were first incubated in nutrient broth in an incubator with a temperature of 37 $^\circ{\rm C}$ and a shaking rate of 150 rpm for 24 h. Then, using a spectrophotometer, their optical density (OD) at 600 nm was adjusted to 1 by adding sterile nutrient broth. After that, autoclaved nutrient agar was poured into 10-cm diameter petri dishes and cooled to be gelled. Finally, 0.1 ml of the bacteria solution was poured onto each petri dish, followed by putting the circular samples on them. The plates containing scaffolds were put into 37 $^\circ{\rm C}$ incubators for 24 h, and the thickness of the inhibition zone around the scaffolds was measured to evaluate the antibacterial activity of the specimens.

It is worth mentioning that the scaffolds were soaked in PBS for 30 min before putting in the petri dishes, and their excess fluid was removed using filter papers. This test was carried out three times for each specimen, and the results are presented as average $\pm$ standard error.

#### Cell viability

The MTT cell viability test was used as an indicator of the cell compatibility of the scaffolds. In this method, 3-(4,5-dimethylthiazol-2-yl)-2,5-diphenyltetrazolium bromide or MTT, which is a soluble yellow dye, undergoes the mitochondrial reductase in living cells and reduces to an insoluble purple dye called formazan or (E,Z)-5-(4,5-dimethylthiazol-2-yl)-1,3-diphenylformazan. This dye remains inside the cells until being extracted by a solvent called DMSO. Finally, the optical density (OD) of the obtained purple solution is evaluated by a spectrophotometer to investigate the scaffolds’ cell compatibility.

In the present study, the specimens, which were in 24 well plates, were first sterilized by being soaked in ethanol 70% overnight and then undergoing UV exposure for two hours (one hour for each side). Then, they were washed three times (15 min each time) by sterile PBS. In the following, the MTT assay was done using two types of cells: L929 cells (50,000 cells/sample, passage 5) and adipose-derived stem cells (20,000 cells/${\mathrm{cm}}^{2}$, passage 4–6).

The MTT assay for L929 cells was done on days 1, 3, and 5 after culturing the cells on the scaffolds in a DMEM cell culture medium containing 10% FBS. The test was performed for the adipose-derived stem cells on days 1, 4, 7, and 14 after cell culture in a DMEM/F12 cell culture medium containing 10% FBS. Besides, the cells cultured on the adhesive well plates were used as controls in the present experiment. To measure the samples’ OD, a BioTek ELISA plate reader (ELx800) was utilized, and the test was performed three times per sample. The results are reported in average $\pm$ standard error.

Finally, the cell viability percentage of the samples was calculated using Eq. (11).

11 $$\mathrm{Cell \,viability }(\mathrm{\%}) = \frac{OD\, of \,the \,sample}{OD\, of \,the\, control\, group} \times 100.$$

#### Cell adhesion

To study the cells’ morphology cultured on the scaffolds, SEM imaging was used. To this end, the previously described cells, L929 cells (50,000 cells/sample, passage 5) and adipose-derived stem cells (20,000 cells/${\mathrm{cm}}^{2}$, passage 4–6) were used. Glutaraldehyde was used to fix the cells in specific time intervals, and after washing the samples by PBS, dehydration of the cells was done utilizing differents ethanol solutions (in six 15-min steps starting from ethanol 50% to ethanols 100%).

The SEM images of the samples containing L929 cells were taken on days 1 and 5 after cell culture using a Seron AIS2300C device. The FE-SEM images were taken on days 1 and 7 after cell culture using a TeScan—Mira III device for the ones with adipose-derived stem cells.

#### Statistical analysis

The results are analyzed statistically to investigate significant differences among different samples. To this end, the one-way ANalysis Of VAriance (ANOVA) method followed by Tukey’s honestly significant difference (HSD) post hoc tets is used. The statistical analysis is performed by SPSS software, version 20.0, and the P-value of less than 0.05 is considered the significant difference between the specimens.

Besides, the results are reported as average $\pm$ standard error. The standard error ($SE$) is calculated according to Eq. (12), in which $SD$ and $n$ are standard deviation and the sample size, respectively.

12 $$SE= \frac{SD}{\sqrt{n}}.$$

## Results and discussion

### Morphology

**Figure 1:**
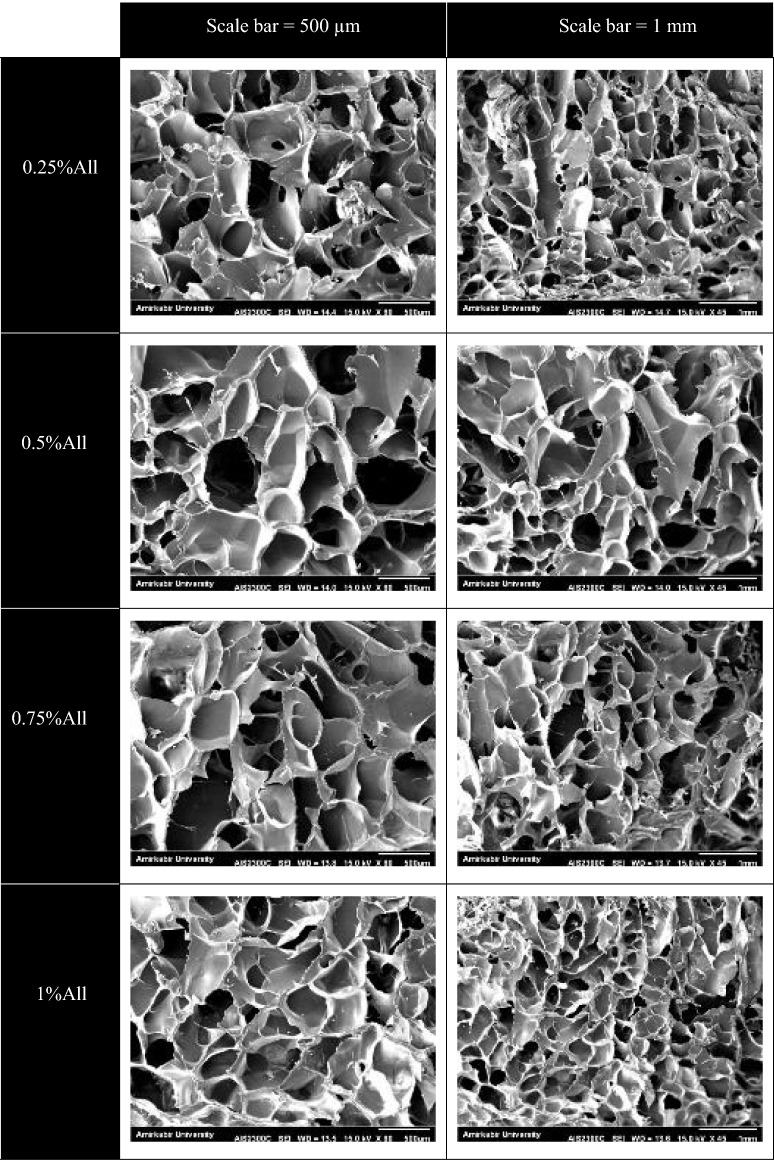
SEM images of the cross-section of the samples. For each sample, two different images in terms of magnification are provided. The scale bars in the left and right images are 500 µm and 1 mm, respectively.

The fabricated scaffolds were light yellow and opaque. To investigate the samples’ microscopic structure and measure their average pore size and porosity, SEM images of the samples’ cross-sections were analyzed, as shown in Fig. 1. The average pore size of a scaffold is a crucial factor in cell penetration and proliferation inside it^32^. The SEM image analysis using Image-J software indicated that the samples’ mean pore size was in the range of 390–460 µm for different specimens, shown in Table 1. As seen in Fig. 1, the samples contain interconnected open pores, an essential parameter for exchanging gas, waste, and nutrients for cells inside the scaffold^33^. Among the fabricated scaffolds, the most uniform structure belongs to the sample with 1% allantoin. It is also worth mentioning that some granules were observed on the surface of the samples. Such granules, which were believed to be from allantoin, chitosan, or impurities, were also found on chitosan films containing allantoin in a previous study^34^.

**Table 1:** The percentage of porosity and the average pore size of the hydrogels.

Sample	Pore size (average $\pm$ SE)	Porosity (by SEM) (%)	Porosity (by ethanol displacement) (%)
0.25%All	396.84 $\pm$ 29.36	63.1 $\pm$ 1.8	27.9 $\pm$ 1.5
0.5%All	435.53 $\pm$ 31.38	62.6 $\pm$ 1.1	30.7 $\pm$ 2.8
0.75%All	458.66 $\pm$ 32.17	58.0 $\pm$ 0.2	29.2 $\pm$ 1.3
1%All	441.00 $\pm$ 24.88	61.6 $\pm$ 0.9	31.9 $\pm$ 3.2

The porosity is measured by two methods: analyzing SEM images and the ethanol displacement technique. The results are presented in average $\pm$ standard error.

Although the mean pore size of the created samples is close to the chitosan–gelatin-based skin scaffolds of several studies^35,36^, it is higher than the skin scaffolds’ average pore size in most studies. Some investigators have reported that the average pore size in the range of 100–200 µm is recommended for skin tissue engineering^37,38^. The formation of large pores in the present scaffolds might be due to both gelatin and chitosan hydrophilicity, resulting in forming large ice crystals in the freeze-drying method^39^. The pore size in the freeze-drying process also depends on parameters such as solution concentration, cross-linking degree, and cooling rate^40^. Altering these factors can modify the presented scaffolds’ structure.

Porosity highly affects the biological and mechanical characteristics of a scaffold^41^. It plays a vital role in cell migration and proliferation, and its amount influences the gas and nutrients exchange, especially when the vascular system does not function properly^32^. The scaffolds’ porosity obtained by SEM image analysis was between 57 and 64%, as indicated in Table 1, which is comparable to some other studies^14,41,42^. However, the porosity values obtained from the ethanol displacement method were relatively lower than the ones measured by analyzing SEM images. As indicated in Table 1, the scaffolds’ porosity was between 27 and 32% using the ethanol displacement technique. The results also showed that the specimen containing 0.75% allantoin had the largest average pore size (458.66 $\pm$ 32.17 µm), but low porosity percentage (58.0 $\pm$ 0.2% by SEM image analysis and 29.2 $\pm$ 1.3 by the ethanol displacement technique). On the other hand, the sample with 0.25% allantoin showed the least mean pore size (396.84 $\pm$ 29.36 µm). However, the statistical analysis suggested no significant difference among the samples in terms of mean pore size and porosity percentage (*p* > 0.05).

### PBS absorption behavior

**Figure 2:**
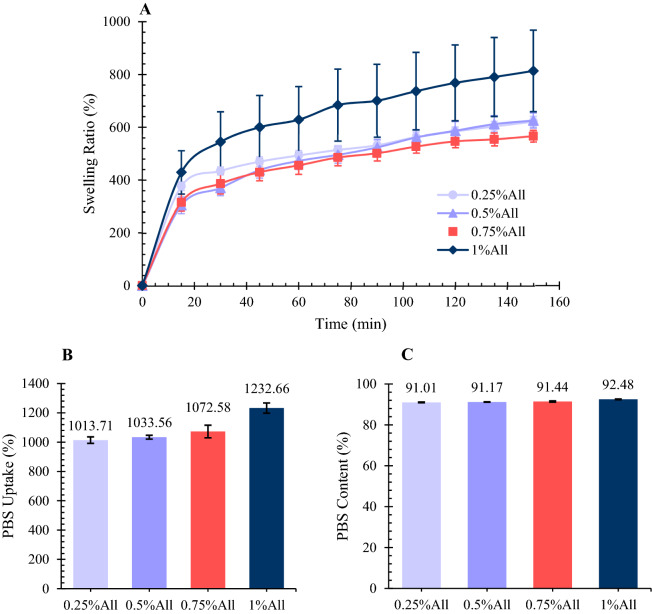
(**A**) The swelling ratio of the samples in PBS. According to the results, the samples absorb PBS around five to seven times their dry weights in 150 min. (**B**) The PBS uptake ability of the samples. (**C**) The PBS content of the specimens after being soaked in PBS for 24 h. The results are presented in average $\pm$ standard error (n = 3).

Traditional wound dressings were used to keep the wound site dry and defending the body against microorganisms^7^. However, a moist environment is proven to result in a faster and more effective healing process^7,43^. It improves the ingredients exchange into the wound, reduces pain, decreases bacterial contamination, and lessens scar formation^17^. Therefore, an ideal skin scaffold should absorb wound exudates and keep the wound moist^29^. The crucial factors determining a scaffold’s fluid absorption behavior include polarity, chemical structure, porosity, cross-linking degree, and so on^44^. In the present study, the swelling ratio of the scaffolds in 150 min, as well as their fluid uptake and fluid content in 24 h, has been measured, as shown in Fig. 2.

The swelling ratio of the samples is presented in Fig. 2A. According to the results, the scaffolds gained PBS 2.9 to 3.7 times their weight (mean of SR(%) ≈ 292–377%) in the first 15 min of the experiment. The slope of the swelling ratio diagrams decreased in time, and the samples absorbed PBS 5.5 to 6.5 times their weight at the end of the 150 min of the swelling ration test (mean of SR(%) ≈ 566–626%). This trend was comparable to several previous studies in which the swelling ratio of their gelatin/chitosan-based scaffolds was from 300 to 6000% in 150 min^14,45,46^. The specimen containing 1% allantoin showed the highest ability to absorb PBS; however, the statistical analysis suggested no significant difference between the different samples (*p* > 0.05).

The PBS uptake of the specimens in 24 h is reported in Fig. 2B. The scaffolds’ average PBS uptake amount was from 1013 to 1233%, based on the results. The sample with 1% allantoin showed the maximum PBS uptake ability; however, no significant difference was observed according to the statistical analysis (*p* > 0.05). The scaffolds’ PBS content was also measured after being soaked in PBS for 24 h, as indicated in Fig. 2C. The PBS content of the swollen scaffolds varied from 91 to 92%. According to the results, the skin scaffolds containing allantoin are able to absorb high amounts of fluid, which is a huge benefit for skin tissue engineering applications. They are able to absorb the wound exudate and keep the wound area moist. The samples’ porous structure and the hydrophilic groups in both gelatin and chitosan, such as –OH and –NH2 groups, result in scaffolds’ high PBS absorption^47,48^.

### Gel fraction

As the cross-linking density affects other properties of a scaffold, such as degradation rate, mechanical properties, and so on, it is essential to examine it. The density of crosslinking in polymer hydrogels can be relatively measured using simple absorption tests or gelation calculations of the samples. Low crosslinking leading to decrement of gelation will cause higher water uptake ability; whereas, the water uptake capacity of the scaffolds will decrease when crosslinking, and thus the gelation value increases, restricting the possibility of swelling. Herein, the gelation fraction of the scaffolds has been determined as a criterion of cross-linking density.

**Figure 3:**
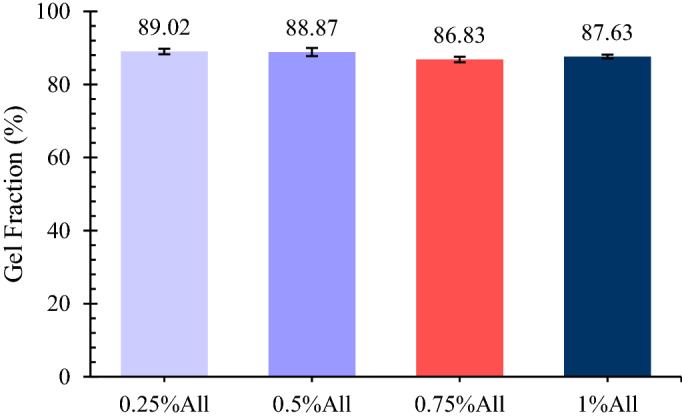
The gel fraction of the specimens. The results are presented in average $\pm$ standard error (n = 3).

The gel fraction for the fabricated samples is indicated in Fig. 3. According to the results, the scaffolds’ gel fraction varies between 86 and 89%, with no significant difference among them (*p* > 0.05). In the present study, the cross-linking occurs mainly by use of EDC, a zero-length cross-linker^49^. In the case of chitosan and gelatin combinations, EDC covalently links the carboxylic acid groups of gelatin to the –NH_2_ groups of its own structure or chitosan^50^. As allantoin also contains –NH_2_ groups, EDC is able to bind it to the carboxylic groups of gelatin covalently.

### Apparent density

**Figure 4:**
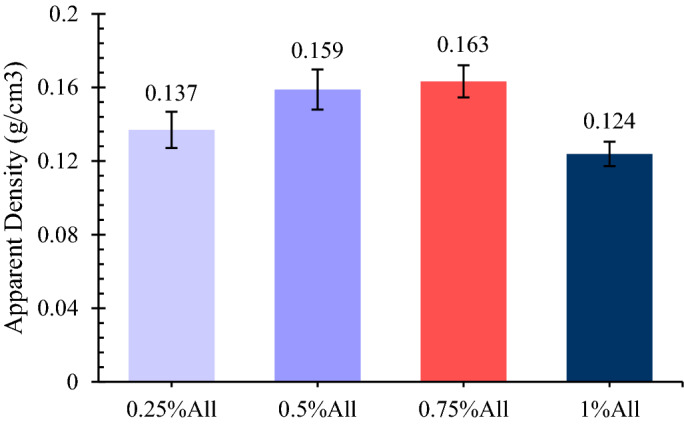
The apparent density of the samples. The results are presented in average $\pm$ standard error (n = 3).

The apparent density of the samples is reported in Fig. 4. The sample containing 0.75% allantoin presents the highest apparent density, which might be due to its lowest porosity among other specimens. The scaffolds’ average apparent density varies between 0.124 and 0.163 g/cm^3^, with no statistically significant difference among them (*p* > 0.05). This result is consistent with a previous study in which chitosan/gelatin hydrogel was fabricated^45^. However, other researchers reported lower apparent density for their chitosan/gelatin-based scaffolds, which can be due to the lower polymer amount in their initial solutions^36^.

### Degradation rate

**Figure 5:**
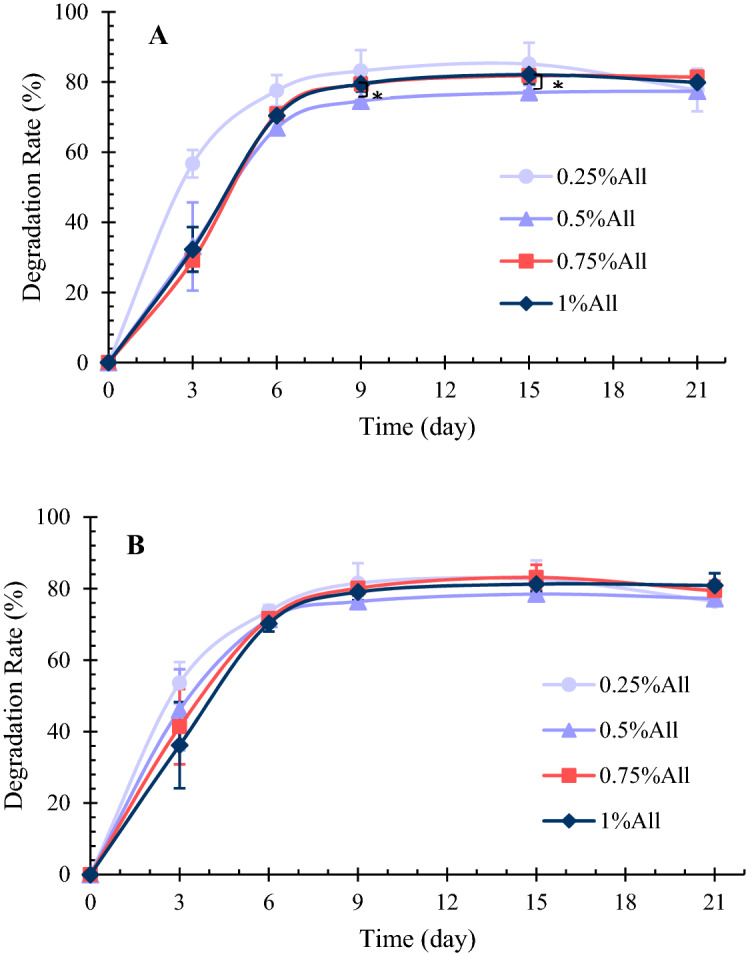
(**A**) The degradation rate of the specimens in PBS and 37 $^\circ{\rm C}$. The sample containing 0.5% allantoin degrades significantly slower than the one with 1% allantoin on the 9th and the 15th days of the experiment. (**B**) The degradation rate of the specimens in PBS containing lysozyme and 37 $^\circ{\rm C}$. *Indicates a statistically significant difference between the samples (*p* < 0.05). The results are presented in average $\pm$ standard error (n = 3).

The degradation rate of a scaffold should be proportional to the tissue regeneration rate. In the case of skin tissue engineering, the scaffold should not fully degrade prior to completing the wound healing’s proliferation stage, which is about 3 weeks^45^. Therefore, in the present study, the samples’ degradation rate is investigated in 21 days in PBS and PBS containing lysozyme, as shown in Fig. 5. Lysozyme is the principal enzyme for degrading chitosan in in-vivo conditions, such as the wound healing process^21,51^.

According to the degradation results in PBS (Fig. 5A), all the samples except the one containing 0.25% allantoin lost about 30% of their weights in 3 days. Their degradation rate remained almost the same during the next 3 days of the experiment. These specimens’ degradation rate decreased after the sixth day of the test and became almost zero after the ninth day. The scaffold with 0.25% allantoin lost about 55% of its weight during the first 3 days of the experiment, but its degradation rate reduced over time, similar to the degradation pattern of other samples. After 21 days of being soaked in PBS and 37 $^\circ{\rm C}$, the samples lost about 80% of their weights, with no significant difference between different specimens (*p* > 0.05). The samples’ disintegration started in the middle parts, and after 21 days, their remnants were in ring shapes.

A similar degradation pattern was observed for the degradation test in PBS containing lysozyme (Fig. 5B). The most considerable difference between the two conditions was found on the third day of the experiment, in which the degradation of the samples was greater in PBS with lysozyme, except for the 0.25% allantoin sample. On the third day of the degradation test in PBS containing lysozyme, increasing the amount of allantoin resulted in decreasing the degradation; however, the difference among the specimens was not significant (*p* > 0.05), and such a pattern was not observed in the other intervals of the experiment. Although the degradation rate in the lysozyme solution was higher than the PBS solution at first, they were similar to each other on the following days of the test, and the samples lost about 80% of their weights after 21 days of the experiment using lysozyme solution.

A variety of degradation patterns has been reported for chitosan/gelatin-based skin scaffolds in the literature. The results in the present study are comparable to some of them^39,51^. As mentioned earlier, the samples’ degradation rate reduced over time, and in week two, it was lower than week one, which contradicts some previous studies^25^. Overall, the samples’ degradation rate seems suitable for skin tissue engineering, for they do not fully disintegrate in 3 weeks, yet lose 80% of their weight to be replaced by the newly formed tissue.

### Uniaxial tension

The mechanical properties of skin scaffolds are essential to be evaluated from two main aspects: (1) the scaffolds should have appropriate mechanical characteristics to be appropriately handled and not to be torn while the wound contracts during the healing process and (2) the scaffolds’ mechanical traits, such as stiffness, affect cell functions, such as cell attachment, proliferation, and differentiation^15,52,53^. As suggested, the more the mechanical properties of scaffolds resemble the normal human skin, the better their function will be^52^. If the scaffolds are too rigid, they might result in pain, and if they are too loose, they may fail due to the contraction during the healing process^54^. Therefore, it is essential to analyze skin scaffolds’ tensile properties to confirm a balance between rigidity and flexibility^7^.

Different values obtained by variant methods have been reported in the literature for Young’s modulus (E) of skin, which seems to depend on age and skin location on the body. For instance, Agache et al. analyzed the skin from the back and reported an average Young’s modulus of 0.42 MPa for young people and 0.85 MPa for the elderly^55^. Barel et al. reported an average Young’s modulus of 0.135–0.169 MPa for forearm and 0.206–0.321 MPa for forehead^56^. Zahouani et al. measured Young’s modulus of the arm using the in-vivo indentation method, reporting an average value of 0.0083 MPa^57^. Annaidh et al. reported the values of 83.3 MPa, 21.6 MPa, and 54% for the average amounts of Young’s modulus, ultimate tensile strength, and failure strain for the skin from the back, respectively^58^.

**Figure 6:**
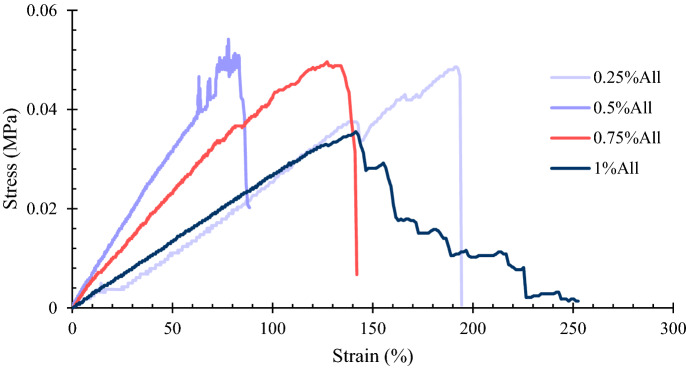
The stress–strain diagram of the tensile test for one of the replications. The sample containing 1% allantoin was gradually ruptured after reaching its maximum tensile strength leading to the shown stress–strain curve.

The results of the uniaxial tension experiment are presented in Fig. 6 and Table 2. Some fluctuations are observed in the stress–strain curves, which might be related to the gradual rupture of polymeric chains. It is also worth mentioning that the sample containing 1% allantoin did not tear suddenly, but ruptured gradually, leading to a step-by-step stress–strain curve descending after reaching its ultimate tensile strength. As shown in Table 2, the specimens’ tensile strength varies between 0.0406 $\pm$ 0.0030 MPa and 0.0992 $\pm$ 0.0230 MPa; their elongation at break is from 81.17 $\pm$ 29.96% to 109.98 $\pm$ 42.14%, and their Young’s modulus is in the range of 0.0654 $\pm$ 0.0207 − 0.1291 $\pm$ 0.0355 MPa. According to the results, the most flexible structure belongs to the sample containing 1% allantoin (E = 0.0654 $\pm$ 0.0207 MPa), while the specimen with 0.5% allantoin possesses the most rigid structure (E = 0.1291 $\pm$ 0.0355 MPa). It seems that adding allantoin to the substrate stiffens it until the concentration of allantoin reaches 0.5%. Adding more than 0.5% of allantoin to the structure appears to decrease the scaffold’s stiffness. It might be due to the fact that allantoin is easily dissolvable in aqueous solutions up to 0.5% at room temperature. To dissolve more than this amount, the temperature must be increased. It is possible that for the samples containing 0.75% and 1% allantoin, some allantoin particles do not dissolve completely, acting as start points for rupture in the tensile test. However, statistical analysis suggested no significant difference among the samples (*p* > 0.05).

**Table 2:** The mechanical properties of the scaffolds resulted from the uniaxial tensile experiment.

Sample	Tensile strength (MPa)	Elongation at break (%)	Young’s modulus (MPa)
0.25%All	0.0799 $\pm$ 0.0170	109.98 $\pm$ 42.14	0.1023 $\pm$ 0.0385
0.5%All	0.0992 $\pm$ 0.0230	82.44 $\pm$ 7.98	0.1291 $\pm$ 0.0355
0.75%All	0.0542 $\pm$ 0.0040	81.17 $\pm$ 29.96	0.0866 $\pm$ 0.0249
1%All	0.0406 $\pm$ 0.0030	109.35 $\pm$ 58.18	0.0654 $\pm$ 0.0207

The results are indicated as average $\pm$ standard error.

Previous studies on fabricating chitosan/gelatin-based scaffolds for skin tissue engineering applications have reported a wide range of mechanical properties for their samples. The average tensile modulus of the scaffolds created by Tseng et al. was reported to be 0.0417 MPa in the wet state^54^. The tensile strength, elongation at break, and elastic modulus of the scaffolds fabricated by Pezeshki-Modarres et al. were in the ranges of 0.072–0.097 MPa, 3–3.8%, and 2.63–3.45 MPa, respectively^59^. The Young’s modulus, tensile strength, and elongation at break for the scaffolds introduced by Angulo et al. were about 0.23–4.99 MPa, 0.004–0.18 MPa, and 3.96–23.52%, respectively^51^. Liu et al. constructed chitosan/gelatin scaffolds with average tensile modulus and maximum tensile strength in ranges of 0.0007–0.0014 MPa and 0.01–0.04 MPa, respectively^36^. The chitosan/gelatin-based scaffolds fabricated by Lu et al. possessed an average Young’s modulus of 0.87 MPa, an average tensile strength of 0.98 MPa, and an average elongation at break of 20.23% in the wet state^46^.

The tensile strength and Young’s modulus of the scaffolds in the present study are comparable to many previous studies on porous chitosan/gelatin-based scaffolds in the literature. Furthermore, their elongations at break are considerably higher than most of the previously fabricated similar skin scaffolds. These differences in mechanical characteristics lie in parameters such as porosity, pore size, pore interconnectivity, the mechanical traits of the polymers, and samples’ fabrication and cross-linking methods. For instance, it is mentioned that increasing the pore size decreases Young’s modulus^54^. The average pore size in the present scaffolds is higher than several previous studies, leading to their lower Young’s modulus. The Young’s moduli of present substrates are comparable to normal human skin in some locations, such as the forearm and arm^56,57^.

### Rheological behavior

**Figure 7:**
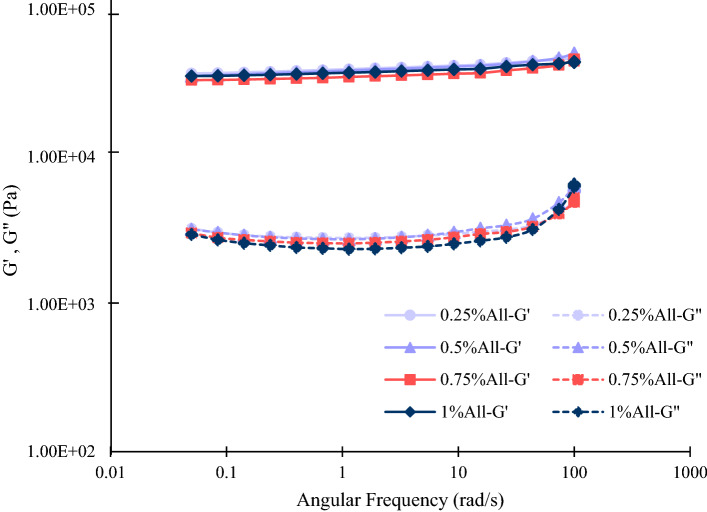
The viscoelastic properties of the specimens resulted from the rheology test. G′ and G′′ represent storage and loss modulus, respectively.

Rheological properties of the stem cells’ microenvironment influence their fate^42,60^. Therefore, it is important to evaluate the rheological characteristics of tissue-engineered scaffolds. To analyze the scaffolds’ rheological behavior, their linear viscoelastic region was first obtained using the strain sweep test. According to the results, for strain rates up to 10%, the viscoelastic behavior of the substrates was linear. Therefore, the frequency sweep tests were performed at a constant strain rate of 5% to ensure that the experiment was done in the linear viscoelastic region. The storage modulus (G′) and the loss modulus (G′′) were then measured for the samples in different frequencies, as indicated in Fig. 7.

Based on the results, there was no significant difference among the different specimens with different amounts of allantoin in terms of the storage modulus and the loss modulus. As indicated in Fig. 7, the storage modulus is considerably greater than the loss modulus for all samples and frequencies. This means that the elastic behavior of the scaffolds dominates their viscous behavior. According to the results, the storage modulus was less sensitive to the frequency changes in comparison to the loss modulus. For instance, for the sample with 1% allantoin, the storage modulus raised from 0.0354 to 0.0438 MPa (an increase of 23.7%) during the frequency sweep test. However, this sample’s loss modulus augmented from 0.00298 MPa to 0.00654 MPa (an increase of 119.5%) during the experiment. It is also worth mentioning that the rise in the loss modulus got sharper for frequencies above 43.8 rad/s.

### Allantoin release profile

**Figure 8:**
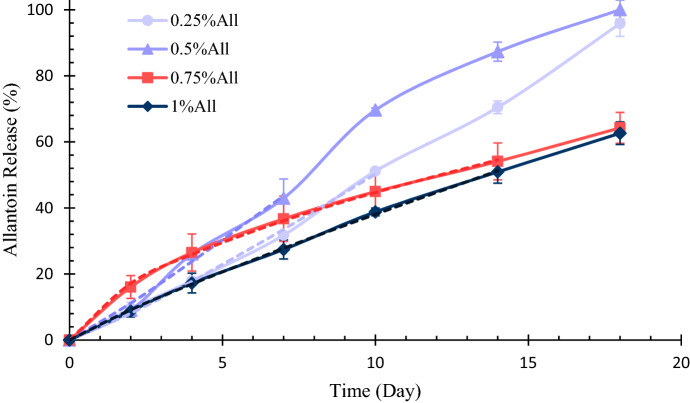
The allantoin release profile from different samples during 18 days (n = 3). To do the release experiment, the specimens were soaked in PBS at 37 $^\circ{\rm C}$. The dashed lines indicate the curve fitting of allantoin release profiles with the Korsmeyer-Peppas equation, whose parameters are presented in Table 3.

The in-vitro drug release profile can be utilized to investigate the effect of the scaffolds on the mechanism and rate of drug release^61^. To study the allantoin release profile from the scaffolds, the standard curve of allantoin was first obtained using a spectrophotometer, which is presented in Fig. S1 in the Appendix. Based on this standard curve, the release profile of allantoin from different samples was obtained as indicated in Fig. 8. According to the results, the samples containing 0.25% and 0.5% allantoin almost released all of their allantoin content in 18 days. The samples with 0.75% and 1% allantoin showed a lower allantoin release rate in general and released about 60% of their allantoin content in 18 days. However, statistical analysis showed no significant difference among the specimens (*p* > 0.05).

The Korsmeyer–Peppas equation was utilized to investigate the mechanism of allantoin release from the samples. The dashed lines in Fig. 8 indicate the curve fitting of allantoin release from the scaffolds using this equation. The parameters obtained from this curve fitting are shown in Table 3. The results suggest that for the samples containing 0.25% and 0.5% allantoin, the diffusion exponent is higher than 1, meaning that the drug release profile follows the super case II transport mechanism, in which the drug release is erosion-controlled^62^.

**Table 3:** The parameters obtained from curve fitting of allantoin release profile with Korsmeyer-Peppas equation.

Sample	K	n	R^2^
0.25%All	3.695	1.134	0.998
0.5%All	5.279	1.086	0.995
0.75%All	11.363	0.595	0.999
1%All	4.995	0.882	1.000

On the other hand, curve fitting of allantoin release from the samples containing 0.75% and 1% allantoin with Korsmeyer–Peppas equation resulted in 0.5 < n < 1, indicating anomalous diffusion, in which the drug release mechanism is both erosion-controlled and diffusion-controlled^31,62^.

According to the results, the allantoin release mechanism for the samples with 0.5% allantoin and less is erosion-controlled, while increasing the amount of allantoin in the scaffolds leads to a combination of diffusion-controlled and erosion-controlled release mechanisms. This observation might be due to the fact that allantoin can be easily solved in aqueous solutions up to 0.5% at room temperature. Furthermore, as discussed earlier, EDC is able to covalently bond allantoin to gelatin because of the presence of –NH_2_ groups in the allantoin structure. Therefore, it seems that most of the allantoin content in the samples with 0.5% allantoin or less, bonds to the chitosan/gelatin scaffold. In this case, allantoin can be released from the substrate by the scaffold’s degradation.

On the other hand, an allantoin content of more than 0.5% cannot be easily solved in aqueous solutions at room temperature. Therefore, in the samples with 0.75% and 1% allantoin, a portion of allantoin content is bonded to the scaffold, while some allantoin particles are just entrapped in the scaffold’s structure, resulting in a combination of diffusion- and erosion-controlled release.

### Antibacterial assessment

Infection is considered one of the main reasons for a delay in the wound healing process, for it lengthens the inflammatory stage and postpones collagen synthesis, prevents epidermal migration, and results in further damage to the tissue^4,7^. Therefore, antibacterial activity is a crucial factor in wound dressings and skin scaffolds design and fabrication. The most significant bacteria in wounds’ contamination are *Staphylococcus aureus* (*S. aureus*), *Escherichia coli* (*E. coli*), and *Pseudomonas aeruginosa*^4^. Hence, in the present study, the antibacterial activity of the scaffolds against *S. aureus* (gram-positive) and *E. coli* (gram-negative) is investigated.

**Figure 9:**
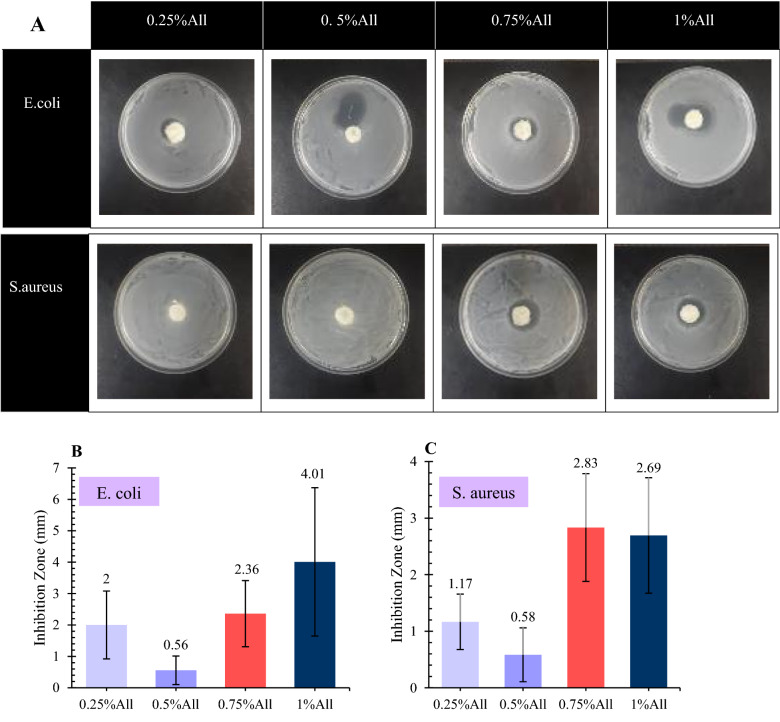
(**A**) The antibacterial assay against *S. aureus* and *E. coli* bacteria. The inhibition zone around the specimens is evident in the presented images. The inhibition zone values around the scaffolds in the antibacterial assay are shown against (**B**) *E. coli* and (**C**) *S. aureus* bacteria. The results are presented in average $\pm$ standard error (n = 3).

The results of the antibacterial assessment are presented in Fig. 9. According to the observations, the bacteria did not grow underneath any of the samples. Besides, on average, all the samples exhibited antibacterial activity against both bacteria. The scaffolds containing 0.75% and 1% allantoin had the highest, and the sample with 0.5% allantoin showed the least antibacterial capacity towards both *S. aureus* and *E. col*i. It is also worth mentioning that the microorganisms’ population in the antibacterial tests is possibly much higher than that in the wound environment, for the body does not provide the optimum situation for bacterial growth^63^. Therefore, even the samples exhibiting the lowest antibacterial capacity might hinder infection in the in-vivo case.

It seems that the specimens owe their antibacterial capability to both chitosan and allantoin. Although some researchers did not observe any antibacterial activity for the drugless chitosan-based scaffolds^37^, most previous studies have demonstrated the antibacterial activity of chitosan^43,64^. Rahman et al. also reported that chitosan’s antibacterial characteristics might be contact-dependent, for it prevented bacterial attachment on its surface while permitting bacterial growth in suspension^48^. As reported in the literature, chitosan’s polycationic structure plays a vital role in its antibacterial capability^20^. The chitosan’s polycations, which are protonated amino groups, interact with the bacteria’s negatively charged surface, leading to their death because of alterations in cells’ permeability and leakage of their internal components^16,46^. Chitosan can also attach to the negatively charged groups on the surface of bacteria and inhibits their RNA synthesis, which prevents their growth^52^.

As aforementioned, the samples containing 0.75% and 1% allantoin exhibited higher antibacterial activity. Therefore, it seems that allantoin also has an antibacterial capacity and plays a role in the antibacterial properties of the present skin scaffolds. This observation is consistent with a previous study in which allantoin improved the antibacterial capability of chitosan–gelatin biocomposite films^29^.

### Cell viability

Cytocompatibility is one of the critical properties of scaffolds, which should be assessed before using them in-vivo^29,65^. To this end, an MTT assay with L929 cells and adipose-derived stem cells (ADSCs) has been employed. L929 cells are mouse fibroblasts, which are selected because of the critical role the fibroblasts play in the wound healing process^29^. ADSCs are also chosen because they can differentiate into several skin cells contributing to the wound healing process^2^.

**Figure 10:**
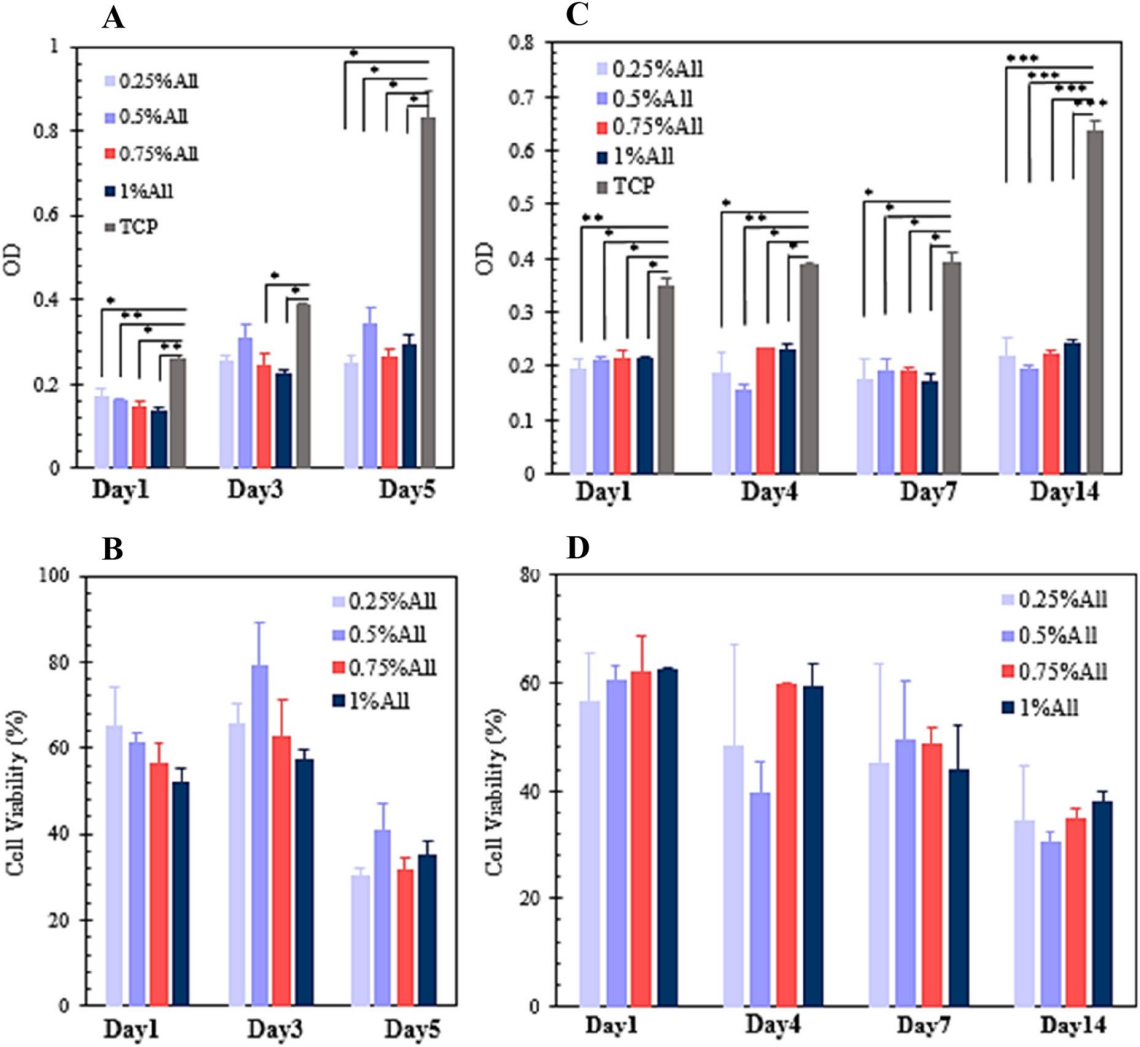
(**A**) The optical density of the MTT assay for L929 cells, (**B**) the L929 cell viability of the samples resulted from the MTT assay, (**C**) the optical density of the MTT assay for ADSCs, (**D**) the ADSC viability of the samples resulted from the MTT assay. The results are presented in average $\pm$ standard error (n = 3).

Figure 10A,B indicate the OD measurements and the consequent cell viability values for the fabricated samples using L929 cells, respectively. According to the results, the OD for all of the specimens increased over time. The sample containing 0.5% allantoin showed higher cell compatibility in comparison to other scaffolds in general, however, not significantly. The control group had higher OD values over the other groups except for the 4th day, in which there was no statistically significant difference between the control and the samples with 0.25% and 0.5% allantoin.

The L929 cell viability for the samples is presented in Fig. 10B, based on which the cell viability for all of the scaffolds increased from the 1st day (about 52–65%) to the 3rd day (about 58–79%). However, the cell viability on the 5th day decreased, despite the fact that the cells could proliferate on the specimens according to the OD measurements. The reason for this observation is that although the OD augmented from the 3rd to the 5th day for the scaffolds, its increase for the control group during this time was considerably higher, meaning that the cells were able to proliferate faster in the control group. As the cell viability amount was calculated based on the OD value of the control, its amount became lower for the 5th day of the experiment, for the difference among the OD of the samples and control was greater on the 5th day than the 3rd day, leading to a lower cell viability value.

Figure 10C,D demonstrate the OD measurements and the cell viability values for the ADSCs, respectively. Based on the results, the scaffolds’ OD values did not change significantly over time. This variable remained almost constant for the control group except for the 14th day of the experiment, in which the control’s OD value increased considerably. Therefore, the cell viability percentage of the scaffolds possessed its lowest amount on the 14th day of the assessment.

These observations indicate that although the L929 cells and ADSCs were able to survive and grow on the scaffolds, their proliferation rates were not as high as the control group. These results are surprising since both chitosan and gelatin are biocompatible polymers, and many previous studies have found the chitosan/gelatin scaffolds highly cytocompatible^41,63,66^. Sakthiguru et al. also reported cell viability above 80% for their chitosan/gelatin allantoin films^29^. The low cell viability of the presented scaffolds may lie in their considerably large pore size, average porosity, and initial high degradation rate. It is also previously mentioned that high ratios of chitosan in a scaffold inhibit fibroblasts’ migration, decreasing cell proliferation^59^.

### Cell adhesion

**Figure 11:**
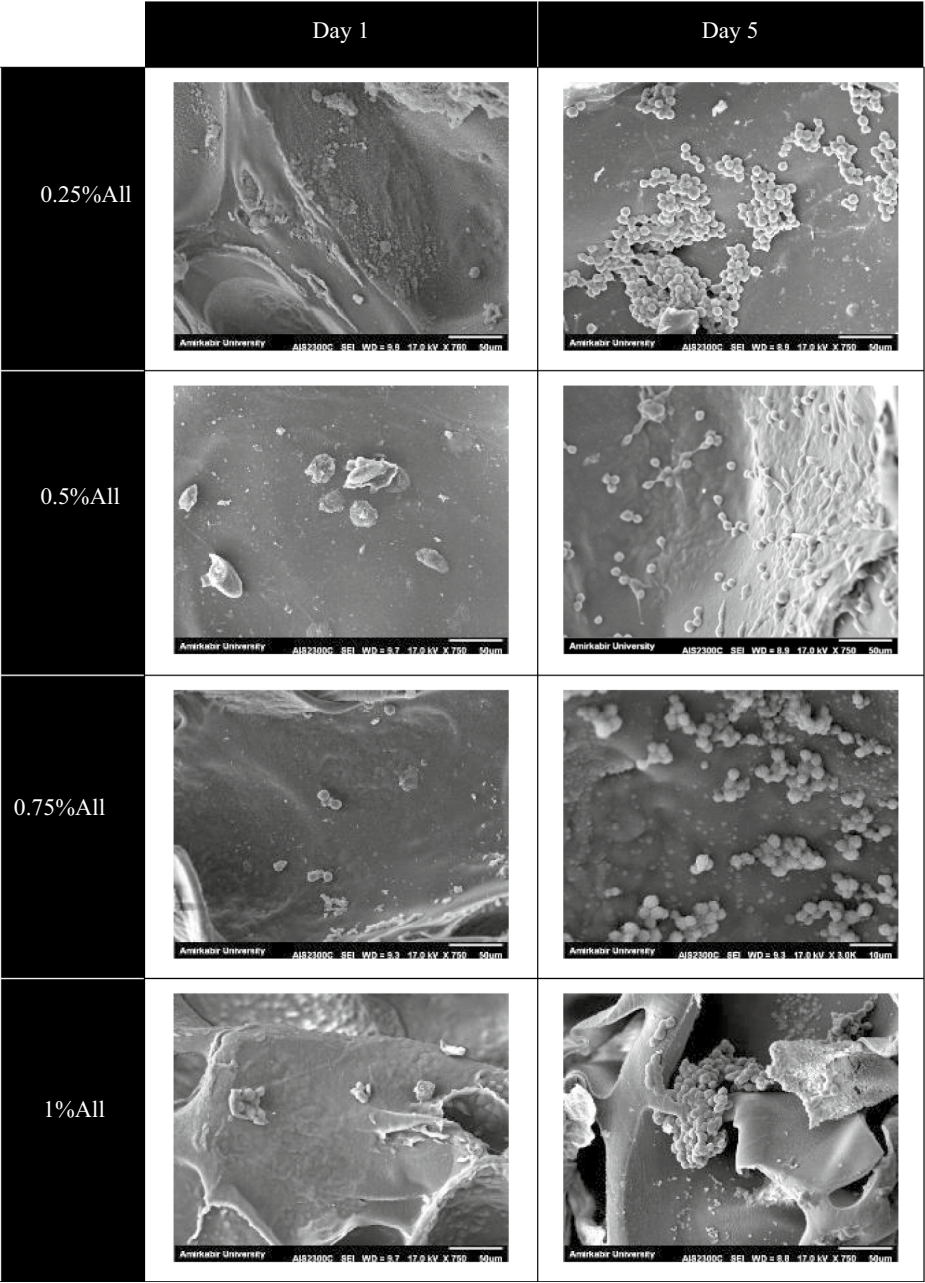
The SEM images of L929 cells cultured on the scaffolds containing different amounts of allantoin. The left and right photos belong to the first and the fifth day after cell culture, respectively. The scale bars in all the images represent 50 µm except for the 0.75%All sample at day 5, in which the scale bar shows 10 µm.

To investigate the cell attachment and morphology after seeding on the scaffolds, SEM imaging was utilized. The two aforementioned cell types, L929 cells and ADSCs, were used in this regard. Figure 11 demonstrates the SEM images of L929 cells on the samples after 1 and 5 days of cell culture. Based on the images, the cells adhered to the samples and increased considerably after 5 days. L929 cells were able to penetrate into the pores and distribute inside the scaffolds, which is an essential factor in cell compatibility and final success in using the scaffold in-vivo.

L929 cells possessed spherical shapes on the samples except for the one containing 0.5% allantoin, in which most of the cells could appropriately spread on the surface and into the pores of the scaffold. The condition in which the cells do not flatten and hold their spherical morphology is called passive adhesion^67^, the case for the specimens with 0.25%, 0.75%, and 1% allantoin. On the contrary, the sample with 0.5% allantoin allowed the cells to flatten well on its surface, called active adhesion^67^. Hence, it seems that the scaffold with 0.5% allantoin provides the best condition for fibroblast attachment.

**Figure 12:**
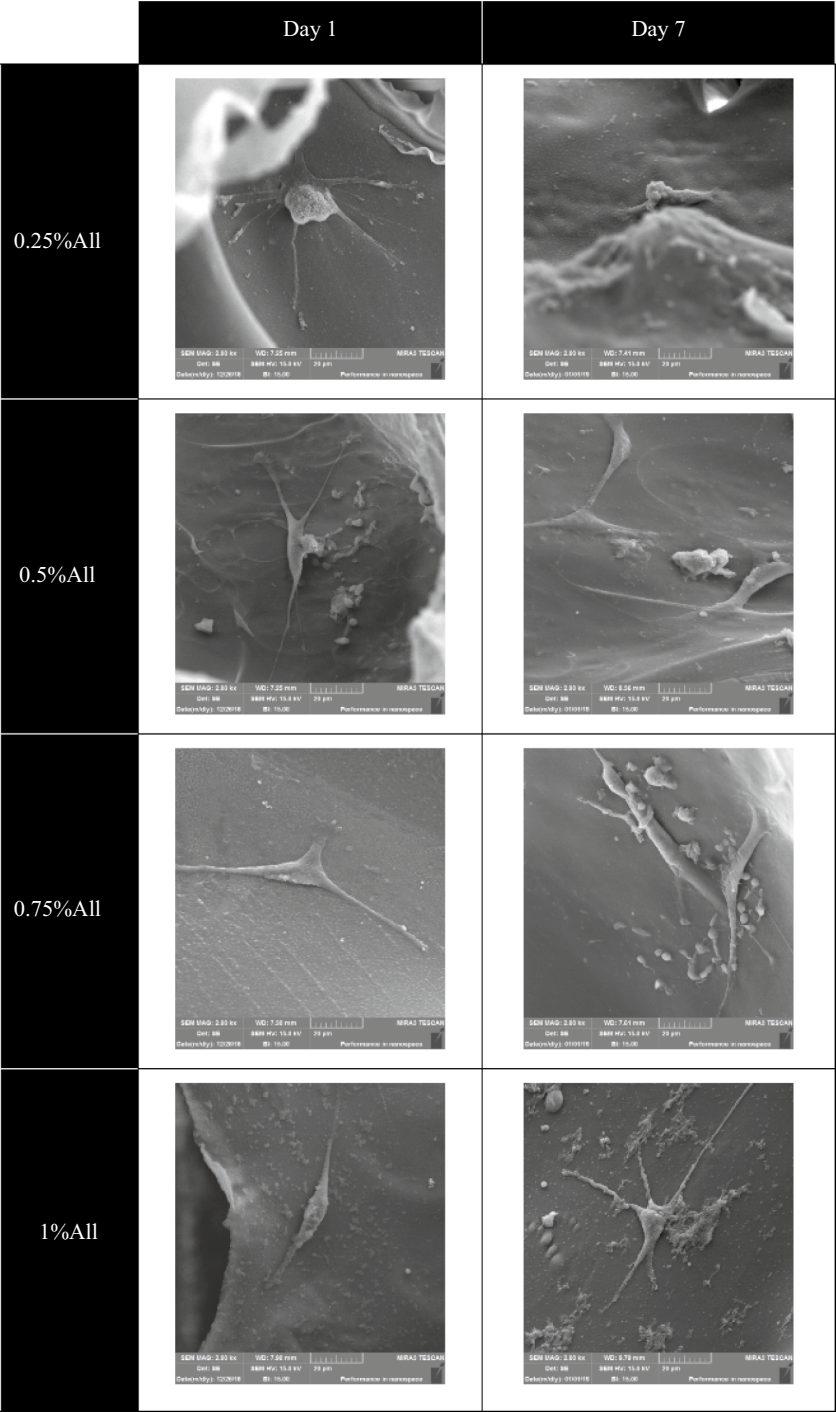
The FE-SEM images of ADSCs cultured on the scaffolds containing different amounts of allantoin after one and seven days of cell culture. The scale bars represent 20 µm.

Figure 12 represents the ADSCs on the scaffolds after 1 and 7 days of cell culture. Another set of FE-SEM images of ADSCs cultured on the scaffolds with a different image magnification is also presented in Fig. S2 in the Appendix. According to the observations, the ADSCs were able to adhere and spread appropriately on all scaffolds. ADSCs also could penetrate into the pores and possess branched morphology. The ADSCs’ active adhesion on the samples indicates their favorable properties for cells to attach and grow. As observed in Fig. 12, it seems that ECM appeared around the cells after seven days of cell culture.

The fabricated scaffolds owe their suitable cell attachment properties to the hydrophilicity of their structure, as well as the unique characteristics of chitosan and gelatin. Chitosan contains positively charged groups, which is favorable for the cells to interact electrostatically due to the presence of negative charges on the cells’ surface^37,68^. Gelatin also plays a vital role in cell attachment because of the presence of RGD in its structure^29^. RGD is an amino acid sequence that plays a significant role in cell adhesion^59^.

## Conclusion

Chitosan/gelatin-based scaffolds containing four different amounts of allantoin were constructed and underwent in-vitro characterization in the present study. To the authors’ knowledge, it was the first time to employ allantoin in a 3D porous structure of a skin scaffold. Several experiments were utilized to discover the in-vitro properties of the fabricated scaffolds. SEM imaging revealed that the samples possessed an average large pore size (390–440 µm) and relatively low porosity (about 58–63%). The scaffolds were able to absorb high amounts of PBS due to the hydrophilicity of the involved polymers, i.e., chitosan and gelatin. The PBS uptake of the sample with 1% allantoin reached 1233% after being soaked in PBS for 24 h, which was the highest among the specimens. The scaffolds’ gel fractions were between 86 and 89%, and they possessed apparent densities in the range of 0.124–0.163 g/cm^3^. The samples lost almost 70% of their weights during the first six days of the degradation assays in PBS and PBS with lysozyme; however, their degradation rate decreased, leading to maintaining about 20% of the samples after three weeks of the experiment.

The uniaxial tension assessment revealed the high flexible structure of the scaffolds, especially the ones with 0.75% and 1% allantoin. The Young’s modulus, tensile strength, and elongation at break of the samples were in the range of 0.0654–0.1291 MPa, 0.0406–0.0992 MPa, and 81.17–109.98%, respectively. The rheology test demonstrated that the storage modulus for all of the samples was higher than their loss modulus, suggesting that the scaffolds’ elastic behavior dominates their viscous behavior. The allantoin release profile assessments showed that the samples with 0.25% and 0.5% allantoin released almost all of their allantoin content in 18 days and followed an erosion-controlled release mechanism. On the other hand, the specimens with 0.75% and 1% allantoin released almost 60% of their allantoin content in 18 days and followed a combination of erosion- and diffusion-controlled release mechanisms.

All of the specimens exhibited antibacterial activity against *S. aureus* and *E. coli* bacteria. The specimens with 0.75% and 1% allantoin showed greater inhibition zones against the aforementioned bacteria, suggesting that the samples’ antibacterial activity was related to both chitosan and allantoin. The MTT assay showed that the cells were able to proliferate on the scaffolds, however, not as fast as their proliferation on the control group. The cell attachment analysis indicated that the scaffolds had favorable properties for ADSCs and L929s attachment. In most assessments, there was no statistically significant difference among the different samples. All in all, the present study suggests that the fabricated scaffolds possess exciting characteristics, such as antibacterial behavior, making them appropriate for skin scaffolds. However, additional modifications should be employed to make their microstructure and biocompatibility closer to an ideal skin scaffold.

## Supplementary Information

Supplementary Information.
