# Supplementary material for: Fabrication and characterization of scaffolds containing different amounts of allantoin for skin tissue engineering
Source: Sci Rep. 2021 Aug 9;11:16164. doi: 10.1038/s41598-021-95763-4 (PMC8352935; doi:10.1038/s41598-021-95763-4)
Supplement: Supplementary file 1 — Supplementary Information. [file 41598_2021_95763_MOESM1_ESM.docx]

**In-vitro characterization of chitosan/gelatin-based scaffolds containing different amounts of allantoin with an application in skin tissue engineering**

Yeganeh Dorri Nokoorani^1^, Amir Shamloo^1*^, Maedeh Bahadoran^1^, Hamideh Moravvej^2*^

1. Mechanical Department, Sharif University of Technology, Tehran, Iran

2. Skin Research Center, Shahid Beheshti University of Medical Sciences, Tehran, Iran

**Appendix**

Fig S1. Calibration curve for allantoin at 210 nm

| Day 7 | Day 1 |  |
| --- | --- | --- |
| 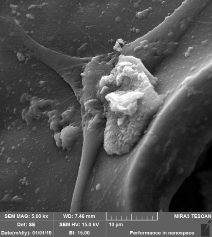 | 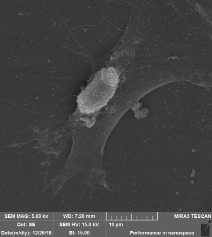 | 0.25%All |
| 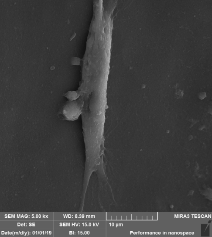 | 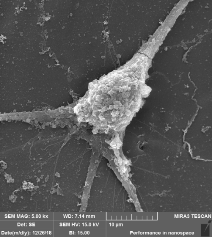 | 0.5%All |
| 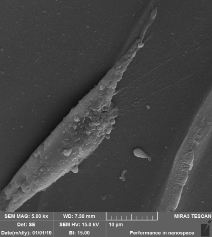 | 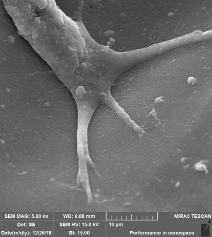 | 0.75%All |
| 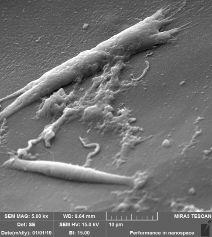 | 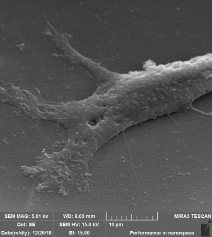 | 1%All |

Fig S2. The FE-SEM images of ADSCs cultured on the scaffolds containing different amounts of allantoin after one and seven days of cell culture. The scale bars represent 10 µm.
